# Genetic Influence on Extended-Release Naltrexone Treatment Outcomes in Patients with Opioid Use Disorder: An Exploratory Study

**DOI:** 10.3390/brainsci16010023

**Published:** 2025-12-24

**Authors:** Farid Juya, Kristin Klemmetsby Solli, Ann-Christin Sannes, Bente Weimand, Johannes Gjerstad, Lars Tanum, Jon Mordal

**Affiliations:** 1 Division of Mental Health and Addiction, Vestfold Hospital Trust, 3103 Tønsberg, Norway; 2 Faculty of Medicine, University of Oslo, 0313 Oslo, Norway; 3 Department of R&D in Mental Health, Akershus University Hospital, 1478 Lørenskog, Norway; 4 Norwegian Centre for Addiction Research, University of Oslo, 0313 Oslo, Norway; 5 Faculty of Health Sciences, Oslo Metropolitan University, 0176 Oslo, Norway; 6 Center for Mental Health and Substance Abuse, University of South-Eastern Norway, 3045 Drammen, Norway; 7 School of Health Sciences, Kristiania University College, 0107 Oslo, Norway

**Keywords:** opioid use disorder, genetic polymorphisms, COMT, OPRM_1_, antagonist treatment, treatment outcomes

## Abstract

**Background/Objectives**: The variation in the treatment outcomes of extended-release naltrexone (XR-NTX) including the potential role of genetic factors are poorly understood. This study aimed to explore the potential association between the catechol-O-methyltransferase (COMT) rs4680 and mu-opioid receptor (OPRM_1_) rs1799971 genotypes and XR-NTX treatment outcomes in patients with opioid use disorder (OUD) specifically focusing on treatment retention, relapse to opioids, number of days of opioid use, and opioid cravings. **Methods**: This was a 24-week, open-label clinical prospective, exploratory study involving patients with OUD who chose treatment with monthly injections of intramuscular XR-NTX. Men and women aged 18–65 years with OUD according to the Diagnostic and Statistical Manual of Mental Disorders, Fifth Edition, were included. The participants were interviewed using the European Addiction Severity Index. Survival analyses and linear mixed models were used to analyze the data. **Results**: Of the 162 participants included in this study, 138 (21% female) initiated treatment with XR-NTX, with 88 genotyped for COMT rs4680 and 86 for OPRM_1_ rs1799971. Heterozygous Met/Val carriers of COMT rs4680 were less likely to relapse to opioids compared with those with the COMT rs4680 Met/Met genotype. No significant association was observed for the OPRM_1_ polymorphism. **Conclusions**: Patients with the COMT rs4680 Met/Val genotype exhibit a reduced risk of relapse to opioids and may therefore derive greater benefit from XR-NTX treatment compared with those with the COMT rs4680 Met/Met genotype. Future studies should be conducted with a larger number of participants and possibly include other genetic variants and treatment outcomes. The trial is registered at ClinicalTrials.gov (#NCT03647774) and the EU Clinical Trial Register (#2017-004706-18).

## 1. Introduction

In the past decade, extended-release naltrexone (XR-NTX), an opioid receptor antagonist, has emerged as a viable addition to treatment options for patients seeking abstinence from opioids, and may provide care to patients who were not previously enrolled in opioid maintenance treatment [1,2,3]. XR-NTX can be used in various settings, including inpatient and outpatient treatment facilities, primary care offices, emergency departments and correctional facilities [4,5,6]. It may improve treatment compliance through monthly injections and is reported to be effective in preventing relapse and reducing opioid use [3,4]. However, not all individuals respond equally to XR-NTX treatment. The psychosocial factors predicting a positive response to this treatment have not yet been established [4]. Moreover, the association between genetic factors and treatment outcomes remains relatively unexplored in patients with opioid use disorder (OUD) being treated with XR-NTX.

Among the genetic factors that could be associated with OUD and treatment outcomes are variations in the genes encoding the catechol-O-methyltransferase (COMT) enzyme and the mu-opioid receptor (OPRM_1_). Specifically, the functional and nonsynonymous rs4680 single-nucleotide polymorphism (SNP) and rs1799971 SNP may be of particular importance [5]. The COMT rs4680 SNP results in the substitution of the amino acid valine (Val) with methionine (Met), where the Met allele exhibits three to four times less enzyme activity [6]. This can increase dopamine levels in the prefrontal cortex, potentially influencing the response to opioid treatment [7]. Thus, the major allele G (Val) of COMT rs4680 may be linked to more frequent relapse to heroin in patients with OUD [8]. Additionally, the same allele is associated with higher dropout rates from treatment compared with A (Met) carriers in opioid agonist treatment (OAT) [9].

OPRM_1_ rs1799971 has been extensively studied in association with OUD [10]. The SNP rs1799971 A > G, located in exon 1 of the OPRM_1_ gene, induces a substitution of the amino acid asparagine (Asn) with aspartic acid (Asp) at the 40th amino acid residue (Asn40Asp) [11]. Earlier observations have suggested that the OPRM_1_ rs1799971 SNP may affect treatment outcomes in patients with OUD, particularly in response to methadone treatment [12,13]. The minor allele G (Asp) is associated with prolonged heroin abstinence in patients with OUD [14]. However, a recent study found no significant interaction between four opioid-related genes (including OPRM_1_ rs1799971) and relapse or retention with daily sublingual buprenorphine–naloxone versus monthly XR-NTX [15]. Among other suggestions for future studies, the investigation of additional gene variants was proposed.

Given the important role of the dopaminergic and opioidergic genes in OUD, the present study aimed to explore possible associations between COMT rs4680 and OPRM_1_ rs1799971 genotypes and XR-NTX treatment outcomes in patients with OUD, specifically regarding treatment retention, relapse to opioids, number of days with opioid use, and opioid cravings.

## 2. Materials and Methods

### 2.1. Design

This exploratory study was part of a larger 24-week, open-label clinical prospective cohort study of monthly injections of XR-NTX with an optional 28-week follow-up (NaltRec study), which is described in detail by Weimand et al. [16].

### 2.2. Participants and Setting

Participants were recruited from addiction clinics in five urban hospitals in Norway, including both inpatient and outpatient centers. Eligible participants were men and women aged 18–65 years with OUD according to the Diagnostic and Statistical Manual of Mental Disorders, Fifth Edition, criteria. The exclusion criteria included alcohol use disorder or serious somatic (e.g., liver failure) or psychiatric (e.g., psychosis) illnesses, or the need for intensive medical treatment that would clearly interfere with study participation. Women of childbearing potential were required to confirm they were not pregnant or lactating and agree to use effective birth control if receiving study medication. The MINI International Neuropsychiatric Interview was used to screen for psychiatric disorders, and a medical doctor examined participants for serious somatic diseases [17]. Recruitment took place from September 2018 to September 2020.

### 2.3. Study Interventions

Screened eligible participants who sought treatment with XR-NTX were referred to an inpatient unit for medically managed withdrawal and included in this study on the day of admission. After induction, they were discharged and followed up in an outpatient setting every 4 weeks, during which they received an XR-NTX injection and completed the study interviews. Through enrollment in the OAT program, all participants could attend counseling if needed and access pharmacological treatment if they withdrew from XR-NTX.

### 2.4. Measurements

Data on demographic characteristics, ethnicity, family history, and treatment outcomes were collected at baseline using the European Addiction Severity Index by trained researchers [18]. Treatment retention was defined as the number of weeks participants remained in treatment and the number of participants completing the study treatment period. Relapse to opioids was defined as the use of any opioids for 7 consecutive days or at least 1 day per week for 4 consecutive weeks. Missing data on opioid use was interpreted as a relapse episode. Data on days of opioid use were collected through interviews using the timeline follow-back technique [19]. Opioid cravings were measured by a two-item ordinal scale that ranged from 0 to 10 and was adapted to the following statement and question: *I need heroin* (0  = “Strongly disagree” and 10 = “Strongly agree”), which assessed present-moment cravings, and *How much or how often did I want and need heroin in the past four weeks?* (0  = “Not at all” and 10  = “Constantly”). The craving item was derived from previous studies that have reported that a rating of the statement I need heroin seems to have a higher extent of validity than other formulations [2,20].

### 2.5. Genotyping

During baseline assessments, the study participants who consented to genotyping were instructed to provide a saliva sample for genotyping in accordance with the manufacturer’s instructions (OrageneRNA sample collection kits, DNA Genotek Inc., Kanata, ON, Canada). Genotyping was conducted using a predesigned TaqMan SNP assay (Applied Biosystems, Foster City, CA, USA). Approximately 10 ng of genomic DNA was amplified in a 5 µL reaction mixture in a 384-well plate containing 1x TaqMan genotyping master mix (Applied Biosystems) and 1× assay mix, the latter containing the respective primers and probes. The probes were labeled with the reporter dyes FAM or VIC to distinguish between the two alleles. In accordance with procedures used in earlier studies, an ABI 7900HT sequence detection system was used [21,22]. Negative controls were included in every run. Approximately 10% of the samples were re-genotyped, yielding a concordance rate of 100%.

### 2.6. Statistical Analyses

No power analysis was performed due to the exploratory nature of this trial, and being part of a larger 52-week study [16]. Descriptive statistics were used to summarize the study sample. Chi-squared tests were conducted to assess whether each genetic variant conformed to Hardy–Weinberg equilibrium [23]. To evaluate treatment retention and relapse to opioids, survival analyses were performed separately for the COMT rs4680 and OPRM_1_ rs1799971 SNPs, with Kaplan–Meier plots illustrating the data. To investigate days of opioid use and opioid cravings, separate linear mixed models were used for each SNP. The OPRM_1_ rs1799971 SNP was dichotomized owing to the low frequency of G carriers (AA vs. G*). The analyses were performed using STATA/SE version 16.0 (StataCorp, College Station, TX, USA). A *p*-value < 0.05 was considered statistically significant.

### 2.7. Research Ethics

Ethical approval for the NaltRec trial, which includes the present study, was granted by the Norwegian Regional Committees for Medical and Health Research Ethics of South East Norway (#2018/132), by the Personal Data Protection representative for each site, and by the Norwegian Medicines Agency [16]. The trial is registered at ClinicalTrials.gov (#NCT03647774) and the European Union Clinical Trials Register (#2017-004706-18). All participants provided written informed consent for their participation. The study treatment was provided free of charge, and participants received no payment or economic compensation for their participation, except for reimbursement of travel expenses if public transportation was used.

## 3. Results

**Figure 1:**
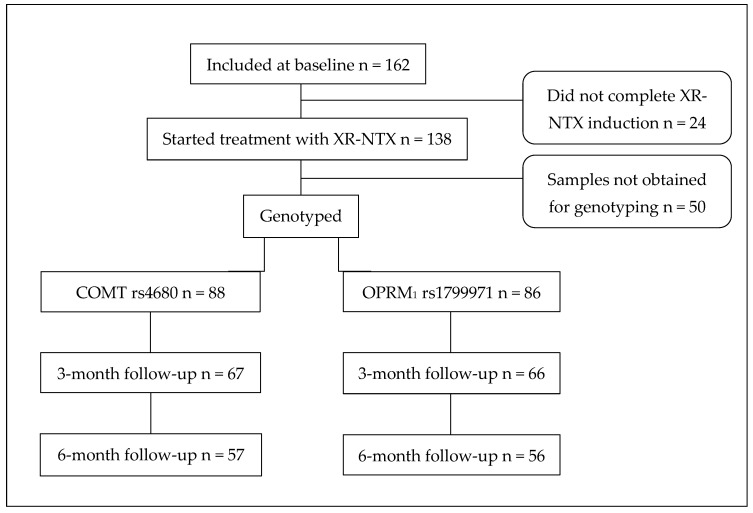
Flowchart for participants receiving XR-NTX who were genotyped for both COMT rs4680 and OPRM_1_ rs1799971 SNPs. XR-NTX—extended-release naltrexone; SNP—single-nucleotide polymorphism.

Of 162 participants included at the baseline, 138 initiated treatment with XR-NTX, of whom 88 were genotyped for the COMT rs4680 SNP and 86 for the OPRM_1_ rs1799971 SNP (Figure 1). For 50 participants, saliva samples were not obtained owing to retraction of consent, participants being unreachable, hyposalivation, or unusable samples due to contamination (mostly from tobacco snuff). At the 6-month follow-up, the number of participants was 57 for the COMT rs4680 SNP and 56 for the OPRM_1_ rs1799971 SNP.

The mean age of the 138 participants who initiated treatment with XR-NTX was 37.8 years (SD = 9.4), 21.0% were female, and 94.9% were North European Caucasians. Participants who were genotyped were compared with those who were not genotyped regarding baseline demographic characteristics, ethnicity, opioid use, opioid cravings, and significant parental psychiatric problems and substance use (Table 1). Sex was the only variable that differed significantly between the two cohorts (*p* = 0.046). Both the COMT rs4680 and OPRM_1_ rs1799971 variants were consistent with Hardy–Weinberg equilibrium (*p* = 0.952 and 0.890, respectively).

### 3.1. Treatment Retention and Association with COMT rs4680 and OPRM_1_ rs1799971

Survival analysis investigating treatment retention included 83 participants with the COMT rs4680 SNP (23 Met/Met carriers, 40 Met/Val carriers, and 20 Val/Val carriers). The mean times in this study for these genotypes were 18.8, 20.8, and 19.8 weeks, respectively. Fourteen (60.9%) of the Met/Met carriers completed the study treatment period of 24 weeks, as did 31 (77.5%) of the Met/Val carriers and 14 (70.0%) of the Val/Val carriers. No statistically significant difference was observed between the genotypes (Supplementary Figure S1).

The analysis regarding treatment retention for the OPRM_1_ rs1799971 SNP included 82 participants (64 AA carriers and 18 G* carriers). The mean times in this study were 20.3 weeks for the AA carriers and 19.3 weeks for the G* carriers. Forty-seven (73.4%) of the AA carriers and 12 (66.7%) of the G* carriers completed the study treatment period of 24 weeks. No statistically significant difference was observed between the AA and G genotypes (Supplementary Figure S1).

### 3.2. Relapse in Association with COMT rs4680 and OPRM_1_ rs1799971

**Figure 2:**
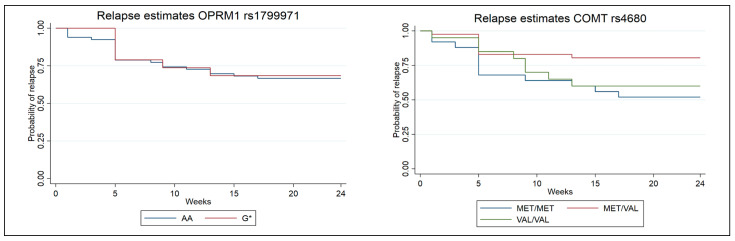
Kaplan–Meier survival estimates for relapse stratified by the different genotypes. COMT rs4680 (*p* = 0.024) and OPRM_1_ rs1799971 (*p* = 0.827).

The survival analyses investigating relapse to opioids included 88 participants with the COMT rs4680 SNP (24 Met/Met carriers, 44 Met/Val carriers, and 20 Val/Val carriers). After testing for equality of survival functions using a log-rank test, a statistically significant difference between genotypes was found (*p* = 0.043). A Kaplan–Meier plot was created to illustrate the data (Figure 2), showing that Met/Val carriers were less likely to relapse to opioids compared with the reference group of Met/Met carriers (hazard ratio = 0.353, *p* = 0.024). No statistically significant difference was observed between the Val/Val and Met/Met genotypes.

A total of 86 participants (67 AA carriers and 19 G* carriers) were successfully genotyped and included in the survival analysis of the OPRM_1_ rs1799971 SNP. A Kaplan–Meier plot illustrated the data (Figure 2). No statistically significant difference was observed between the AA and G genotypes.

### 3.3. Days of Opioid Use in Association with COMT rs4680 and OPRM_1_ rs1799971

No statistically significant interaction effect was observed for the COMT rs4680 genotype; however, an insignificant trend was noted, with COMT Val/Val carriers having fewer days of opioid use (*p* = 0.075) (Table 2). For the OPRM_1_ rs1799971 genotype, no statistically significant interaction effect between AA and G carriers was observed (Supplementary Table S1).

### 3.4. Opioid Cravings in Association with COMT rs4680 and OPRM_1_ rs1799971

No statistically significant interaction effects were observed for the COMT rs4680 or the OPRM_1_ rs1799971 SNPs regarding opioid cravings (Supplementary Table S2A,B).

## 4. Discussion

To the best of our knowledge, this is the first study to address the effect of the COMT rs4680 and OPRM_1_ rs1799971 genotypes on treatment outcomes in patients with OUD who were treated with XR-NTX in an outpatient setting over a 24-week period. Based on our exploratory analyses, heterozygous Met/Val carriers of COMT rs4680 were less likely to relapse to opioids compared with the reference group of Met/Met carriers. Regarding days of opioid use, COMT Val/Val carriers had the fewest days of opioid use, but this trend was not statistically significant. For retention and opioid cravings during treatment, no statistically significant differences were observed between different variants of the COMT rs4680 and OPRM_1_ rs1799971 genotypes.

Interestingly, a link between the Val/Met variant of COMT rs4680 and relapse to opioids were observed. This finding is in contrast with a previous study of heroin-dependent patients in China [8], where the COMT rs4680 Val allele was linked to more frequent heroin relapses. Moreover, our results align with the varying findings of an earlier systematic review on the impact of OPRM_1_ genetic polymorphisms on methadone maintenance treatment [12]. Also, consistent with earlier studies in patients with OUD, we found that genetic variations in the COMT rs4680 and OPRM_1_ rs1799971 SNPs had a minor influence on retention in treatment and opioid cravings in patients with OUD treated with XR-NTX [15].

The overall high retention rate, low frequency of relapse, and decreasing number of days of opioid use over time suggest that XR-NTX treatment was a regimen to which participants were highly committed, regardless of genetic variations in the COMT rs4680 and OPRM_1_ rs1799971 SNPs. Although XR-NTX is not yet available for clinical use in Norway, retention rates tend to be higher in prospective studies than in studies based on clinical journal reviews [1]. However, factors such as the participant-study personnel relationship, differences in follow-up efforts, and variations in expertise may also affect retention rates [24].

Pharmacogenetics is in its early stages and its implementation in clinical care is still limited due to differing views on the quality of the evidence and varying opinions on the clinical usefulness among healthcare professionals [25]. Establishing pharmacogenetic testing as a standard care requires further research, updated guidelines, and collaboration among principal stakeholders [26,27]. The impact of our findings on clinical care is limited to providing information to healthcare professionals about the effective use of XR-NTX and contributing to future studies.

This exploratory study was limited by its relatively small sample size. We included only patients who had initiated treatment with XR-NTX and were genotyped (not intention to treat population) because the genotype effects are expected to rely on physiological interactions between XR-NTX, opioid receptor and enzyme activity. The discrepancy between the number of participants included at baseline and the number of genetic samples obtained was primarily due to the retraction of consent or unavailability of participants for genetic testing, particularly as the last part of data collection occurred during the COVID-19 pandemic. Nonetheless, aside from the borderline sex differences, there were no statistically significant differences in characteristics between participants who were genotyped and those who were not. Thus, it seems that the genotyped participants are likely representative of all participants who initiated treatment with XR-NTX.

### Future Improvements

We recommend that future studies include a larger number of participants for two main reasons. First, as with studies in other fields, genetic studies yield a more reliable and clinically meaningful conclusion with larger sample sizes. Second, given that missing data in longitudinal studies is inevitable, including a larger cohort at baseline is likely to reduce the vulnerability of drop out at the follow-up time points and thus enhance the power of the analyses. This can be achieved by either having a longer recruitment period or collaborating with additional study sites, which would have the added benefit of potentially providing care and support to more patients.

In the present study, a number of saliva samples used for genotyping were contaminated primarily from tobacco snuff, a common form of tobacco in Norway. Higher quality samples could have been obtained through blood samples rather than saliva samples. Although saliva samples are less invasive, the DNA yields from blood samples are higher [28]. Additionally, blood samples have lower levels of DNA contamination [29]. However, this may not be as relevant in countries where tobacco snuff is less used. We also recommend including additional treatment outcome measures beyond treatment retention, relapse to opioids, number of days with opioid use, and opioid cravings. For instance, the Maudsley Addiction Profile measures treatment outcomes across multiple domains, such as substance use, health risk behavior, physical and psychological health, and personal/social functioning [30].

We have explained a reasonable rationale for choosing COMT rs4680 and OPRM_1_ rs1799971 polymorphisms for this study. However, future researchers may consider including other genetic variants related to addiction. For instance, OPRD_1_ SNPs rs2236857 and rs581111 have been reported to be associated with a higher risk of heroin addiction [31].

## 5. Conclusions

Our data suggest that patients with OUD who have the COMT rs4680 Val/Met polymorphism may benefit more from XR-NTX treatment in terms of opioid relapse compared with carriers of COMT rs4680 Met/Met genotype. While the impact of our findings on clinical care is limited, they may contribute to future studies, which should be conducted with a larger number of participants and possibly include other genetic variants and treatment outcomes.

## Figures and Tables

**Table 1:** Baseline demographic characteristics, ethnicity, opioid use, and family history based on cohorts.

	XR-NTX *n* = 138	Genotyped *n* = 88	Not Genotyped *n* = 50	*p*-Value
Age Min, Max Mean (SD)	18, 63 37.8 (9.4)	18, 63 37.4 (9.2)	24, 62 38.8 (9.9)	0.416 ^1^
Sex, *n* (%) Female	29 (21.0)	15 (17.1)	14 (28.0)	0.046 ^2^
Ethnicity, *n* (%) North European Caucasian Other	130 (94.9) 7 (5.1)	84 (95.5) 4 (4.5)	47 (94.0) 3 (6.0)	0.958 ^1^
Age of onset of opioid use, years Min, Max Mean (SD)	10, 39 20.2 (5.5)	10, 39 20.6 (5.6)	10, 33 19.3 (5.3)	0.199 ^1^
Route of Administration Oral Nasal Smoking IV	3 (2.2) 2 (1.4) 26 (18.8) 107 (77.5)	2 (2.3) 2 (2.3) 18 (20.4) 66 (75.0)	1 (2.0) 0 8 (16.0) 39 (78.0)	0.660 ^2^
Opioid use previous four weeks, days Min, Max Mean (SD)	0, 28 18.8 (10.7)	0, 28 18.3 (10.4)	0, 28 20.0 (10.8)	0.450 ^1^
Opioid cravings Present, Mean (SD) Previous four weeks, Mean (SD)	2.81 (3.6) 3.9 (3.9)	2.8 (3.7) 4.3 (3.8)	2.8 (3.6) 3.2 (3.9)	0.976 ^1^ 0.144 ^1^
Significant maternal psychiatric problem, *n* (%) Yes No	41 (29.7) 88 (63.8)	23 (26.1) 59 (67.1)	17 (34.0) 28 (56.0)	0.517 ^2^
Significant paternal psychiatric problem, *n* (%) Yes No	35 (25.4) 85 (61.6)	19 (21.2) 58 (69.9)	15 (30.0) 30 (60.0)	0.421 ^2^
Significant maternal substance use, *n* (%) Yes No	21 (15.2) 110 (79.7)	12 (13.6) 74 (84.1)	10 (20.0) 36 (72.0)	0.299 ^2^
Significant paternal substance use, *n* (%) Yes No	19 (13.8) 106 (76.8)	9 (10.2) 71 (80.1)	9 (18.0) 38 (76.0)	0.430 ^2^

XR-NTX—extended-release naltrexone; *n*—number; SD—standard deviation; ^1^—ANOVA; ^2^—χ^2^-test.

**Table 2:** Estimates of fixed effects parameters from linear mixed models portraying the association between COMT rs4680 and number of days of opioid use at baseline, 3-month and 6-month follow-ups. B—regression coefficient; CI—confidence interval.

Number of Days of Opioid Use in the Previous Four Weeks			
B		95% CI	*p*-Value
Follow-up time points			
Baseline (ref)			
3-month	−21.64	−25.65 to −17.64	0.000
6-month	−20.06	−24.41 to −15.70	0.000
COMT genotype			
Met/Met (ref)			
Met/Val	−2.26	−5.79 to 1.25	0.207
Val/Val	−3.82	−8.02 to 0.38	0.075
Interaction Time × COMT			
Baseline × Met/Met (ref)			
3-month × Met/Val	0.10	−4.86 to 5.08	0.966
3-month × Val/Val	4.99	−1.12 to 11.11	0.109
6-month × Met/Val	−1.46	−6.79 to 3.87	0.591
6-month × Val/Val	2.23	−4.03 to 8.50	0.485

## Data Availability

The NaltRec trial will be finalized in 2025. According to current Norwegian regulations and practice, the data will then be anonymized and deposited in a publicly available data repository (e.g., The Norwegian Centre for Research Data).

## Supplementary Materials

The following supporting information can be downloaded at: https://www.mdpi.com/article/10.3390/brainsci16010023/s1. Supplementary Figure S1. Kaplan–Meier survival estimates for treatment retention stratified by the different genotypes; Figure legend: COMT rs4680 (*p* = 0.293) and OPRM_1_ rs1799971 (*p* = 0.572). Supplementary Table S1. Estimates of fixed effects parameters from linear mixed models portraying the association between the OPRM_1_ rs1799971 genotype and days of opioid use at baseline, 3-month and 6-month follow-ups; Table footer: B—regression coefficient; CI—confidence interval. Supplementary Table S2A. Estimates of fixed effects parameters from linear mixed models portraying the association between COMT rs4680 genotypes and opioid cravings at baseline, 3-month and 6-month follow-ups; Table footer: B—beta coefficient; CI—confidence interval. Supplementary Table S2B. Estimates of fixed effects parameters from linear mixed models portraying the association between the gene variant OPRM_1_ rs1799971 and cravings at baseline, 3-month and 6-month follow-ups; Table footer: B—beta coefficient; CI—confidence interval.
