# Supplementary material for: Genetic Influence on Extended-Release Naltrexone Treatment Outcomes in Patients with Opioid Use Disorder: An Exploratory Study
Source: Brain Sci. 2025 Dec 24;16(1):23. doi: 10.3390/brainsci16010023 (PMC12838570; doi:10.3390/brainsci16010023)
Supplement: Supplementary file 1 [file brainsci-16-00023-s001.zip › Supplementary Figure 1.pdf]

Supplementary Figure 1. Kaplan-Meier survival estimates for treatment retention stratified by the different genotypes

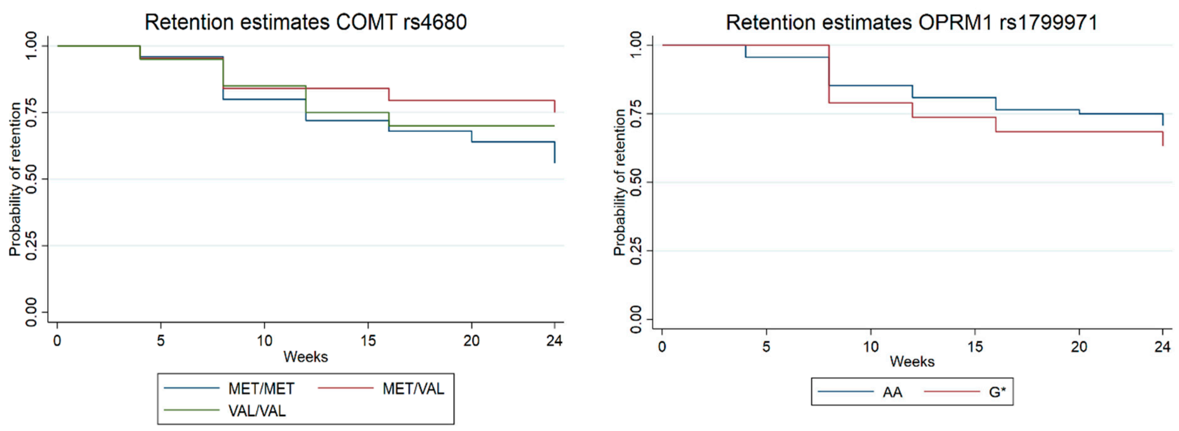

COMT rs4680 (p = 0.293) and OPRM1 rs1799971 (p = 0.572)
