# Supplementary material for: Genetic Influence on Extended-Release Naltrexone Treatment Outcomes in Patients with Opioid Use Disorder: An Exploratory Study
Source: Brain Sci. 2025 Dec 24;16(1):23. doi: 10.3390/brainsci16010023 (PMC12838570; doi:10.3390/brainsci16010023)
Supplement: Supplementary file 1 [file brainsci-16-00023-s001.zip › Supplementary Table 1.pdf]

Supplementary Table 1. Estimates of fixed effects parameters from linear mixed models portraying the association between the OPRM1 rs1799971 genotype and number days of opioid use at baseline, 3-month follow-up, and 6-month follow-up

	**Number of days of opioid use in the previous four weeks**		
	**B**	**95% CI**	***p-value***
**Follow-up time points**			
Baseline (ref)			
Three months	-20.21	-22.65 to -17.76	0.000
Six months	-20.16	-22.69 to -17.63	0.000
**OPRM1 genotype**			
AA (ref)*			
	-1.06	-2.66 to 4.79	0.575
**Interaction Time x OPRM1**			
Baseline x AA (ref)			
Three months x G*	-1.97	-7.32 to 3.37	0.468
Six months x G*	-1.29	-6.92 to 4.34	0.653

B – regression coefficient, CI – confidence interval
