# Supplementary material for: Genetic Influence on Extended-Release Naltrexone Treatment Outcomes in Patients with Opioid Use Disorder: An Exploratory Study
Source: Brain Sci. 2025 Dec 24;16(1):23. doi: 10.3390/brainsci16010023 (PMC12838570; doi:10.3390/brainsci16010023)
Supplement: Supplementary file 1 [file brainsci-16-00023-s001.zip › Supplementary Table 2A.pdf]

Supplementary Table 2A. Estimates of fixed effects parameters from linear mixed models portraying the association between COMT rs4680 genotypes and opioid cravings at baseline, 3-month follow-up, and 6-month follow-up

	Opioid cravings, present			Opioid cravings, previous four weeks		
	**B**	***p-value***	**95% CI**	**B**	***p-value***	**95% CI**
**Follow-up time points**						
Baseline (ref)						
Three months	-2.663	0.000	-4.124 to -1.202	-3.141	0.000	-4.674 to -1.607
Six months	-2.835	0.001	-4.483 to -1.186	-3.116	0.000	-4.821 to -1.412
**COMT rs4680**						
MET/MET (ref)						
MET/VAL	0.686	0.388	-0.873 to 2.246	-0.579	0.377	-1.865 to 0.706
VAL/VAL	-1.110	0.242	-2.970 to 0.750	-0.890	0.255	-2.423 to 0.643
**Interaction effect**						
**COMT x Time**						
Baseline x MET/MET (ref)						
Three months x MET/VAL	-0.793	0.391	-2.606 to 1.019	0.909	0.351	-0.999 to 2.817
Three months x VAL/VAL	1.169	0.291	-1.002 to 3.341	1.944	0.095	-0.340 to 4.228
Six months x MET/VAL	-1.000	0.324	-2.987 to 0.986	0.956	0.364	-1.109 to 3.023
Six months x VAL/VAL	1.838	0.127	-0.524 to 4.200	1.240	0.323	-1.216 to 3.697

B – beta coefficient, CI – confidence interval
