# Supplementary material for: Genetic Influence on Extended-Release Naltrexone Treatment Outcomes in Patients with Opioid Use Disorder: An Exploratory Study
Source: Brain Sci. 2025 Dec 24;16(1):23. doi: 10.3390/brainsci16010023 (PMC12838570; doi:10.3390/brainsci16010023)
Supplement: Supplementary file 1 [file brainsci-16-00023-s001.zip › Supplementary Table 2B.pdf]

Supplementary Table 2B. Estimates of fixed effects parameters from linear mixed models portraying the association between the gene variant OPRM1 rs1799971 and cravings at baseline, 3-month follow-up, and 6-month follow-up

	**Opioid cravings, present**			**Opioid cravings, previous four weeks**		
	**B**	***p-value***	**95% CI**	**B**	***p-value***	**95% CI**
**Follow-up time points**						
Baseline (ref)	-2.750	0.000	-3.638 to -1.861	-2.230	0.000	-3.166 to -1.294
Three months	-2.715	0.000	-3.665 to -1.766	-2.324	0.000	-3.313 to -1.335
Six months						
**OPRM1 rs1799971**						
AA (ref)						
G*	-0.124	0.882	-1.760 to 1.511	-0.428	0.533	-1.772 to 0.916
**Interaction effect OPRM1 x Time**						
Baseline x AA (ref)						
Three months x G*	-0.341	0.735	-2.318 to 1.634	-0.161	0.878	-2.220 to 1.898
Six months x G*	-1.080	0.302	-3.130 to 0.970	-0.139	0.898	-2.264 to 1.985

B – beta coefficient, CI – confidence interval
